# The Comparative Study of WC–Ni Coatings Deposited by APS and HV-APS Processes

**DOI:** 10.3390/ma19132834

**Published:** 2026-07-02

**Authors:** Tadeusz Kubaszek, Anita Slys-Palacz, Marek Goral, Krzysztof Krupa, Marcin Drajewicz

**Affiliations:** 1 Research and Development Laboratory for Aerospace Materials, Rzeszow University of Technology, Zwirki i Wigury 4, 35-029 Rzeszow, Poland; mgoral@prz.edu.pl (M.G.); drajewic@prz.edu.pl (M.D.); 2 Doctoral School of the Rzeszow University of Technology, Powstancow Warszawy 12, 35-029 Rzeszow, Poland

**Keywords:** HV-APS, plasma spray, wear resistance, tribology, porosity

## Abstract

This study investigates the properties of WC–10Ni cermet coatings deposited by plasma spraying using two different plasma torches: a conventional A60 torch (APS) and an advanced Axial III torch (HV-APS). The aim of the work was to compare the microstructure, porosity, surface roughness, phase composition, and mechanical properties (hardness and instrumented indentation), as well as erosion, scratch response, and resistance to tribological wear of the obtained coatings. The coatings were deposited onto S235 steel substrates using WC–10Ni (WOKA 3302) powder. The results revealed that both coatings exhibit a typical lamellar structure characteristic of plasma-sprayed materials; however, distinct differences in surface roughness, porosity, and mechanical response were observed. The coating produced using the Axial III torch showed lower porosity (~6%) and higher hardness (~1000 HV) compared to the coating deposited with the A60 torch (~12% porosity and ~831 HV). Phase analysis confirmed the presence of WC, W_2_C, and Ni in both coatings, indicating partial decarburization of carbides during the spraying process. Erosion resistance tests did not reveal significant differences between the coatings. Erosion testing revealed comparable performance for both coatings, with erosion rates of approximately 0.7 mg/min. Scratch testing showed significantly lower acoustic emission activity for the Axial III coating, indicating less intensive fracture-related events during loading and confirming its more compact microstructure. In contrast, ball-on-disk tribological tests demonstrated comparable wear rates for both coatings (≈9 × 10^−5^ mm^3^·N^−1^·m^−1^), despite the substantially higher hardness of the Axial III coating (1010 HV0.2 compared with 792 HV0.2 for the A60 coating). These results indicate that the improvements in hardness and coating densification achieved by the HV-APS process did not result in a measurable reduction in steady-state sliding wear under the applied test conditions.

## 1. Introduction

Cermet coatings have been widely used for several decades to protect steel surfaces from wear, corrosion, and erosion. Various deposition techniques are employed for their fabrication, including thermal spraying processes such as HVOF (high-velocity oxygen fuel), APS (atmospheric plasma spraying), and detonation spraying, as well as laser cladding and TIG (tungsten inert gas) surfacing [1]. Among these, WC–Ni-based coatings represent a commonly applied group of wear-resistant materials, typically deposited by laser cladding [2,3] or HVOF spraying [4]. Compared with plasma spraying, laser cladding typically provides the benefit of producing coatings with a strong metallurgical bond and very low porosity; however, it also introduces a substantial heat input into the substrate, which can cause thermal distortion and dilution [2,3]. In contrast, HVOF spraying is commonly favored for cermet coatings because its relatively low flame temperature, combined with high particle velocities, tends to limit the decarburization of WC into brittle W_2_C and W phases, thereby enhancing phase stability and coating density [4]. In the case of HVOF, the selection of appropriate process parameters—particularly the particle temperature—plays a critical role in determining the final coating properties [5,6]. Nevertheless, thermal plasma spraying continues to be highly attractive for carbide-based coatings owing to its significantly higher process enthalpy, which allows melting or partial melting of a broader spectrum of feedstock materials, including high-melting-point ceramics and composite powders that are challenging to process using HVOF. These differences provide the basis for selecting appropriate process configurations depending on the required coating characteristics and material system [4].

Despite their technological importance, the available literature on the properties of WC–Ni coatings remains relatively limited. Previous studies have indicated that WC–Ni coatings exhibit lower corrosion resistance compared to coatings with a NiCo matrix [7], although improved corrosion behavior has been reported under specific conditions, such as in the presence of Na_2_S [8]. Furthermore, reduced resistance to cavitation and tribocorrosion has also been observed for these coatings [9,10]. Most of the existing studies on Ni-based matrices focus on coatings produced by alternative techniques, including laser cladding [2,11,12], plasma transfer arc welding [13], and brazing [14]. Various approaches have also been proposed to enhance the performance of WC–Ni coatings, such as the addition of molybdenum to improve high-temperature tribological properties [15] or impregnation with molybdenum disulfide [16].

Cermet WC–Ni coatings constitute a key protective material in sectors demanding high resistance to wear and corrosion [7]. In the petrochemical industry, they are widely applied to pump shafts and shut-off valves operating in aggressive media [2,4]. The mining sector employs these systems to protect the crusher hammers and liners of the crushers against extreme abrasion [7]. In aerospace, they serve as an efficient alternative to hard chromium plating in landing gear actuators and aero-engine components [2,4]. Hydropower and tidal energy implement WC–Ni coatings to protect turbine blades from cavitation and sand-induced erosion [7,9]. In agriculture, these layers drastically reduce the wear of rotary tiller blades and other soil-engaging parts [14]. Furthermore, these materials are applied in automotive brake disks and forming tools subjected to high loads [2,15].

The limited availability of data highlights the need for further investigation of thermally sprayed WC–Ni coatings, particularly those deposited using advanced plasma-based techniques. In recent years, significant progress has been made in plasma spraying technologies, enabling the achievement of deposition conditions comparable to those of HVOF processes [17,18]. These techniques have been successfully applied to a wide range of materials, including ceramic coatings (e.g., Al_2_O_3_ [19], TiO_2_ [20], TiB_2_–SiC [21]), metallic systems (e.g., W/Cu [22]), and conventional wear-resistant cermets such as NiCr–Cr_3_C_2_ [23], as well as more advanced materials [24]. Supersonic plasma spray systems, such as those developed in China [25], have demonstrated the ability to deposit carbide-based coatings, including WC–Co [26] and NiCrBSi [27,28].

Commercial solutions, such as Triplex Pro 200/210 (Oerlikon Metco, Wohlen, Switzerland), also enable the deposition of a wide range of ceramic and cermet coatings, including carbide-containing systems [29,30,31]. A structurally similar device is the Mettech Axial III plasma torch (Mettech, North Vancouver, BC, Canada), which is widely used in Suspension Plasma Spraying (SPS) and solution precursor plasma spraying (SPPS). The Axial III torch, through a dedicated nozzle configuration and axial powder injection into the plasma plume, enables the APS process to be carried out at increased particle velocities, known as the high-velocity atmospheric plasma spraying (HV-APS) process. In contrast to conventional APS systems employing radial powder injection, the axial feeding configuration promotes more uniform particle heating and higher particle acceleration, which may contribute to improved coating homogeneity and deposition efficiency. This configuration has been widely applied in SPS and SPPS processes, particularly for the fabrication of columnar thermal barrier coatings [32,33,34,35,36]. However, there is still a lack of studies that address the application of this torch for the deposition of metal–ceramic coatings, particularly those based on tungsten or chromium carbides [37].

Therefore, the present study proposes the use of the Axial III plasma torch equipped with a supersonic nozzle for the deposition of WC–Ni coatings. The aim is to investigate the feasibility of depositing WC–Ni coatings using the HV-APS process and to evaluate their microstructural, mechanical, and tribological properties, including hardness, scratch behavior, erosion resistance, and sliding wear performance, in comparison with coatings produced by conventional APS.

## 2. Materials and Methods

**Figure 1:**
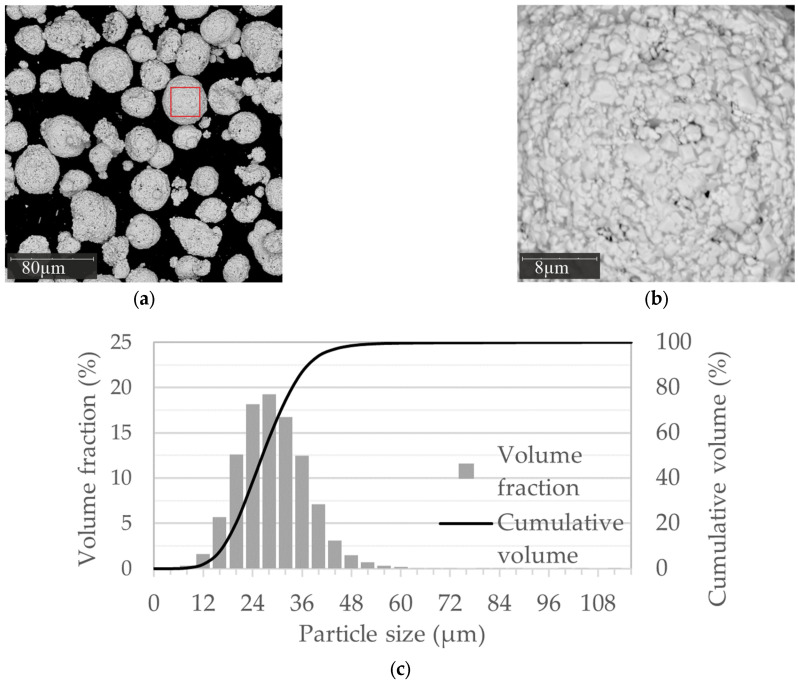
Characteristics of the WOKA 3302 (WC–10Ni) feedstock powder: (**a**) SEM micrograph showing the morphology of the particles at 1000× magnification; (**b**) high-magnification SEM image (9000×) of the area marked with a red frame in (**a**), revealing the surface details of the agglomerated structure; (**c**) particle size distribution (PSD) presented as incremental volume fraction (bars) and cumulative volume (line).

The S235 carbon steel (chemical composition: C: 0.19–0.20 wt.%, Mn: max wt. 1.4%, P: max 0.045 wt.%, N: max 0.012 wt.% (according to EN 10025-2 [38]) was selected as the substrate material, and specimens were prepared in the form of plates with a thickness of 4 mm. WC–10Ni coatings were deposited using WOKA 3302 powder (Oerlikon Metco, Wohlen, Switzerland) with the following nominal chemical composition (according to the manufacturer’s technical data sheet): C: 5.2–6.0 wt.%, Ni: 8.5–11.5 wt.%, Fe < 0.2 wt.%, and W (balance). As shown in Figure 1a, the feedstock exhibits a spheroidal morphology, while high-magnification SEM analysis (Figure 1b) reveals an agglomerated and sintered structure. Although this powder is typically used for HVOF spraying, it was selected to verify its applicability in the HV-APS process. The measured particle size distribution (Figure 1c) is consistent with the manufacturer’s specification (−45 + 15 µm), with precisely calculated characteristic diameters of D10 = 18.56 μm, D50 = 28.14 μm, and D90 = 39.30 μm.

**Figure 2:**
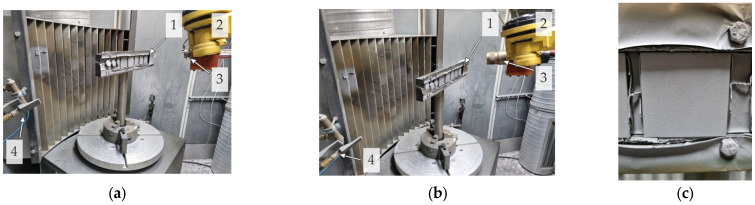
Experimental setup for the plasma spraying process: (**a**) A60 plasma torch (APS); (**b**) Axial III plasma torch (HV-APS); (**c**) macro view of the coated substrate. 1—S235 steel substrate in the holder; 2—industrial robot arm (Fanuc); 3—plasma torch (with respect to the process); 4—compressed air-cooling nozzles.

The spraying processes were performed using a thermal spray system from Thermico GmbH (Dortmund, Germany). The setup was equipped with two types of plasma torches: a conventional single-electrode A60 torch for the APS process and a three-electrode Axial III torch featuring axial powder injection for the HV-APS process. The torches were operated with a Fanuc M710iC industrial robot (Fanuc Corporation, Oshino, Japan), and during coating deposition, the substrate was cooled using compressed air, as illustrated in Figure 2. The spraying parameters were selected based on preliminary studies described in [37,39] and are summarized in Table 1. The two processes differ in terms of plasma gas composition and flow rates, as well as in the configuration of powder injection. In the APS process, the A60 torch features radial powder injection, whereas the Axial III torch in the HV-APS process uses axial powder injection aligned with the plasma plume axis. These distinctions result in different operating conditions for the two spraying systems. For the APS process, an Ar/H_2_ mixture was used (68 and 5 NLPM, respectively), while the HV-APS process relied on an Ar/H_2_/N_2_ mixture with higher overall flow rates (140, 20, and 100 NLPM, respectively). These parameters were chosen according to the documented operating ranges for each torch system described in previous studies [37,39,40,41,42,43].

The morphology of the feedstock powder and the microstructure of the coatings were examined using a Phenom XL scanning electron microscope (SEM) (Thermo Fisher Scientific, Waltham, MA, USA) equipped with an energy-dispersive spectroscopy (EDS) system for chemical analysis. In the analysis, the secondary electron mode (SE) and an electron beam acceleration voltage of 15 kV were applied. Microanalysis of the chemical composition (EDS) was performed on non-etched surfaces.

The particle size distribution of the WOKA 3302 powder was determined using an IPS U analyzer (Kamika Instruments, Warsaw, Poland). The final results were calculated as the average of three measurements. The analysis was performed using 256 measurement classes and an infrared diode light source. Porosity and thickness were evaluated using cross-sectional SEM micrographs by quantitative image analysis.

Porosity was determined from ten random areas captured at a magnification of 2000×, which were manually binarized using Met-Ilo v. 24.01 software (Katowice, Poland) and analyzed with Leica Application Suite v3.7 (Leica Microsystems, Wetzlar, Germany). The average coating thickness was calculated based on 60 individual measurements performed across six random micrographs at a lower magnification of 500×, ensuring a representative assessment of the entire cross-section. All results are reported as average values with calculated standard deviations.

Phase composition analysis for the feedstock powder and the deposited coatings (A60 and Axial III) was performed using an ARL X’Tra X-ray diffractometer (Thermo Fisher Scientific, USA). Measurements were made in the 2θ range of 20 to 80 ° using CuKα radiation with a wavelength of λ = 0.1540562 nm. Phase identification and qualitative analysis were performed using the PDF database provided by the International Centre for Diffraction Data (ICDD).

The surface roughness of all samples was measured using a Surftest SJ-210 (Mitutoyo, Kawasaki, Japan) equipped with SJ-Communication-Tool v. 2.0 software, in accordance with the ISO 21920-2:2021 standard [44]. For each coating variant, three samples were evaluated with five measurements performed on each (15 measurements in total) to determine the mean values and standard deviations of the Ra and Rz parameters.

The mechanical properties were characterized at both the microscale and nanoscale. Microhardness was measured using a Nexus 4303 tester (Innovatest, Maastricht, The Netherlands). The Vickers method was applied with a diamond pyramidal indenter under a load of 1.96 N (HV0.2) maintained for 10 s. Ten measurements were made for each coating variant (five in each of the two selected samples) to ensure statistical reliability. To provide a comprehensive evaluation of the coatings’ mechanical response, instrumented indentation (nanoindentation) was performed using an MHTX tester (Anton Paar TriTec SA (formerly CSM Instruments SA), Corcelles, Switzerland). Measurements were conducted with a Vickers diamond indenter at a maximum load of 2000 mN, with loading and unloading rates of 4000 mN/min and a 10 s pause at maximum load. The Oliver & Pharr method was applied to calculate indentation hardness (H_IT_) and indentation modulus (E_IT_). The adhesion and deformation resistance of the coatings were evaluated using scratch tests using an RST instrument (Anton Paar TriTec SA (formerly CSM Instruments SA), Corcelles, Switzerland). A Rockwell diamond indenter with a radius of 200 µm was used. The tests were carried out in progressive load mode, increasing from 1 N to 200 N over a distance of 10 mm at a constant speed of 10 mm/min. During the tests, normal load (Fn), friction coefficient (COF), frictional force (Ft), and acoustic emission (AE) were recorded, along with penetration depth (Pd). After the tests, the morphology of the scratch tracks was examined using SEM at 1000× magnification to evaluate the failure modes of the coatings and crack propagation.

Erosion resistance tests were conducted according to the ASTM G76-18 [45] standard using a TR-470 air-jet erosion tester (Koehler Instrument Company, Bohemia, NY, USA) at room temperature (25 °C). The samples were prepared as 25 × 25 mm plates. The test duration was set to 10 min with the samples positioned at an impact angle of 90° relative to the nozzle. The abrasive powder was fed at a rate of 2 g/min under an air pressure of 0.2 kg/cm^2^. Mass loss was measured before and after the tests using a KERN ABT 120-4M analytical balance (KERN & SOHN GmbH, Balingen, Germany) with an accuracy of 1 × 10^−4^ g. Macro-scale surface changes and the resulting erosion craters were documented using high-resolution digital macro photography before and after the tests.

Tribological wear resistance was evaluated using the ball-on-disk method using a T-01M tribotester (Łukasiewicz Research Network—ITeE, Radom, Poland) in accordance with the ASTM G99-17 standard [46]. The tests were carried out under a load of 30 N, a linear speed of 0.1 m/s, and a sliding distance of 500 m. A WC ball (10 mm diameter, 1741 ± 5 HV10) was used as the counterbody. During the tests, the friction force was continuously recorded to determine the coefficient of friction (COF). The material volume loss (*V_loss_*) was calculated using the following equation specified in the ASTM G99-17 standard:

(1) $$V_{loss}=2\pi R[r^{2}{sin}^{-1}\left(\frac{d}{2r}\right)-\left(\frac{d}{4}\right)\left(4r^{2}-d^{2}{)}^{\frac{1}{2}}\right]$$

where *R* is the radius of the wear track, mm; *d* is the width of the wear track, mm; and *r* is the radius of the counterbody ball, mm.

The width of the wear track (d) was measured using a Leica DMI3000 M optical microscope (Leica Microsystems, Wetzlar, Germany) in bright-field mode at a magnification of 5×. For each wear track, four randomly selected regions were analyzed, and five individual width measurements were made per image, resulting in a total of 20 measurements per coating type using Leica Application Suite v3.7 software. The average values of d were used for the subsequent calculation of the material volume loss (*V_loss_*). After tribological tests, the wear tracks were examined using SEM to identify the dominant wear mechanisms. SEM observations were performed at 1000× magnification in BSE mode. The wear resistance was quantified based on material volume loss (*V_loss_*) and additionally expressed as a specific wear rate (k), which normalizes the volume loss by the applied load and total sliding distance, allowing for a standardized comparison between the different coating variants; it is defined as

(2) $$k=\frac{V_{loss}}{F\cdot s}$$

where *k* is the specific wear rate, mm^3^·N^−1^·m^−1^; *V_loss_* is the material volume loss, mm^3^; *F* is the applied normal load, N; and *s* is the sliding distance, m.

## 3. Results and Discussion

### 3.1. Microstructure, Porosity, and Chemical and Phase Composition of Coatings

**Figure 3:**
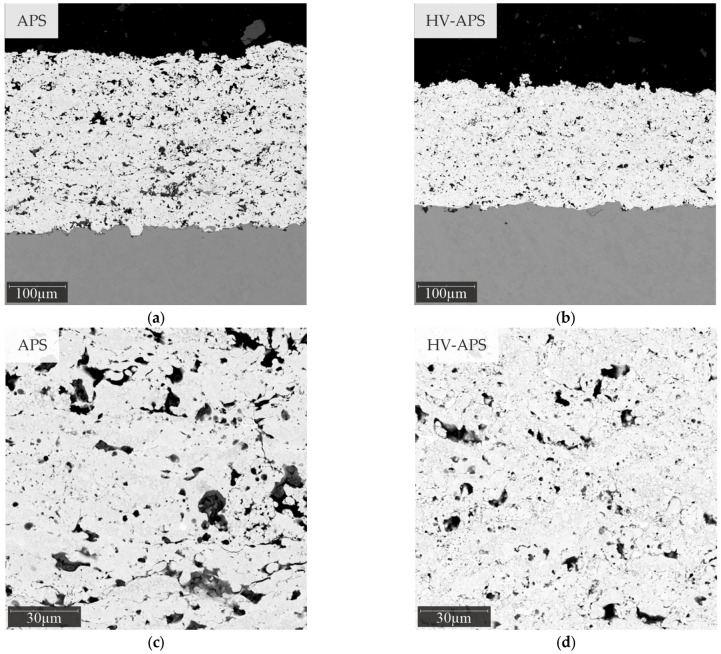
SEM cross-sectional microstructure of the WC–10Ni coating deposited using (**a**,**c**) a conventional A60 plasma torch (APS) and (**b**,**d**) Axial III (HV-APS) plasma torch at different magnifications: (**a**,**b**) 500×; (**c**,**d**) 2000×.

The coatings deposited using the conventional single-electrode A60 torch and the three-electrode Axial III torch exhibit a lamellar microstructure characteristic of atmospheric plasma spraying (APS). In both cases, a distinct banded morphology and the presence of pores are clearly observed (Figure 3). The WC–10Ni coatings demonstrated a relatively uniform thickness, a value of 316.29 ± 10.05 μm for the coating produced with the A60 torch and 204.64 ± 12.34 μm for the HV-APS process using the Axial III torch (Table 2).

Significant differences in porosity were identified (Table 2). The coating produced by conventional APS (A60 torch) exhibited relatively high porosity, reaching nearly 12 vol.%, which is considerably higher than that reported for previously studied composite coatings containing a lower fraction of hard phases (up to 30 wt.%) [40,41], as well as coatings produced from ultrafine carbide powders [42,43]. In contrast, the coating deposited using the HV-APS process (Axial III torch) showed substantially lower porosity, slightly exceeding 6 vol.%. This improvement in coating density may be associated with the much higher overall plasma gas flow rates used in the Axial III system (260 NLPM compared to 73 NLPM for the A60 torch). Together with the axial powder injection setup, these parameters are expected to produce greater particle velocities and more intense in-flight heating, which can lead to increased particle flattening on impact. As a result, the development of a denser and more strongly bonded lamellar structure is promoted. Nevertheless, this value remains higher than that typically achieved by supersonic HVOF spraying [47,48].

**Figure 4:**
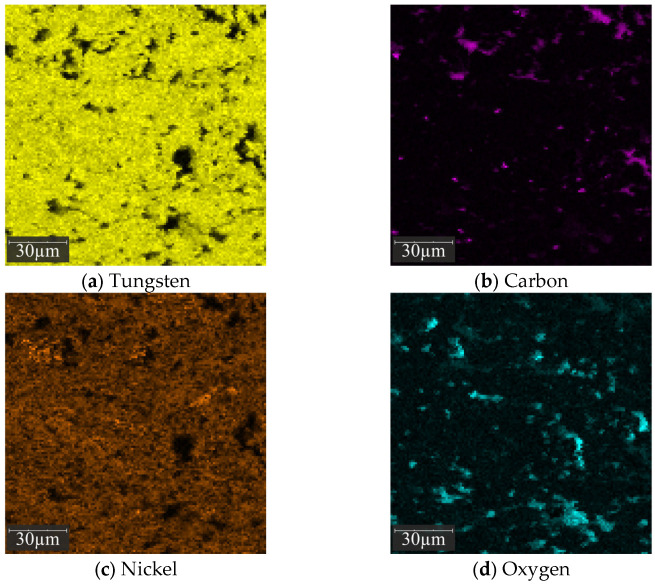
EDS elemental mapping of tungsten (**a**), carbon (**b**), nickel (**c**), and oxygen (**d**) for the WC–10Ni coating deposited by the conventional APS process (A60 torch). The analyzed area corresponds to the high-magnification micrograph presented in Figure 3c.

**Figure 5:**
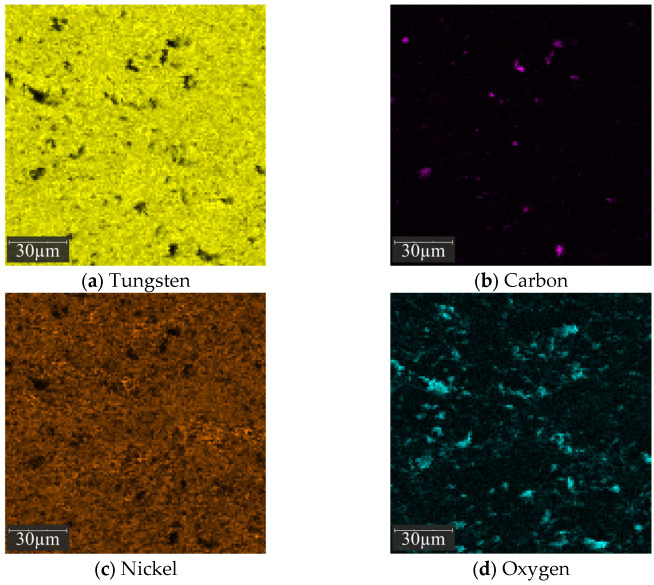
EDS elemental mapping of tungsten (**a**), carbon (**b**), nickel (**c**), and oxygen (**d**) for the WC–10Ni coating deposited by the conventional HV-APS process (Axial III plasma gun). The analyzed area corresponds to the high-magnification micrograph presented in Figure 3d.

Due to the presence of carbon, the chemical composition analysis was performed qualitatively using EDS elemental mapping (Figure 4 and Figure 5). The results indicate that tungsten and carbon are predominantly distributed in regions outside the pores, while nickel, forming the metallic matrix, is uniformly distributed throughout the coating cross-section. Oxygen was detected mainly in porous regions, suggesting localized oxidation phenomena. These observations confirm a relatively homogeneous distribution of carbide phases within the metallic matrix.

**Figure 6:**
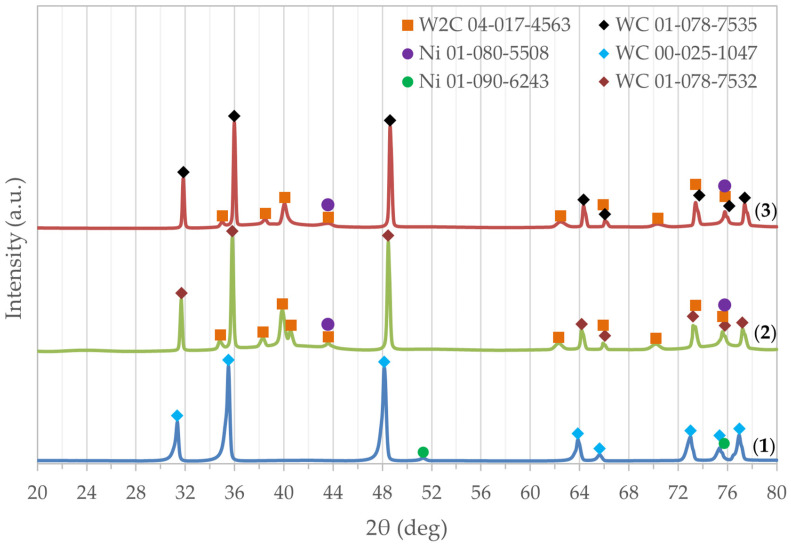
X-ray diffraction pattern of Woka 3302 powder (**1**) and carbide coating produced from Woka 3302 powder using (**2**) Axial III torch (HV-APS) and (**3**) A60 torch (APS).

Phase composition analysis was carried out using X-ray diffraction (XRD) for three samples: the as-received WOKA 3302 powder (Figure 6, pattern 1), the coating obtained by HV-APS (Figure 6, pattern 2), and the coating deposited using the A60 torch (Figure 6, pattern 3). The feedstock powder consisted primarily of two phases: tungsten carbide (WC) and metallic nickel. Although the coatings also contained these primary phases, their diffraction patterns exhibited slight 2θ angular shifts relative to the powder used. These deviations can likely be attributed to substantial residual lattice strain and modifications of the lattice parameters induced by the rapid melting and solidification of particles in the high-energy plasma jet. In order to maximize the accuracy of phase identification and achieve the highest possible Goodness of Match (GOM) values in the ICDD database, different PDF reference cards were used for each coating to better account for their likely differing stress states. Furthermore, the presence of W_2_C was identified in both coatings, indicating partial decarburization of WC during the thermal spraying process. This phase transformation is particularly evident from the appearance of a new diffraction peak at approximately 2θ ≈ 40° in patterns 2 and 3 (Figure 6), which can be attributed to the W_2_C phase. The comparable intensity and position of this peak in both coatings imply that the degree of thermal decomposition of the hard carbide phase is similar in the A60 and Axial III processes, despite differences in torch design and configuration.

The formation of W_2_C is commonly associated with decarburization of WC occurring in the high-temperature environment of thermal spraying processes, where carbon is preferentially lost from the carbide phase, resulting in the formation of carbon-deficient products such as W_2_C and metallic tungsten [49]. The phenomenon of tungsten carbide decarburization during plasma spraying of WC–Ni coatings is a critical process, driven by the extreme temperatures of the plasma jet, which can lead to the decomposition of the hard phase. According to studies by Yuan et al. [6], the degree of WC phase retention in WC–12Ni coatings is strongly correlated with the particle deposition temperature: at temperatures up to 1600 °C, WC retention remains at approximately 86%, but drops sharply to 80% when the temperature rises to 1680 °C. Similar observations concerning carbide degradation and phase evolution in WC-based coatings have also been reported for laser-cladded WC–Ni systems [50]. Decarburization results in the formation of brittle secondary phases, which may adversely affect coating cohesion and mechanical performance. The presence of such phases is generally considered one of the main limitations of high-temperature thermal spraying processes. In contrast, cold spray deposition allows the original carbide phase composition to be largely preserved due to the absence of particle melting and significantly lower process temperatures [51]. The higher thermal input characteristic of plasma spraying therefore promotes phase transformations that are largely suppressed in solid-state deposition processes.

Another contributing factor may be the fine particle size of the WOKA 3302 powder, which is optimized for HVOF spraying and may be more susceptible to thermal decomposition under plasma spraying conditions [52]. Furthermore, HVOF studies have shown that reducing particle overheating is essential for maintaining WC phase stability and minimizing decarburization effects [5].

### 3.2. Surface, Mechanical, and Erosion Properties

The surface and mechanical characteristics of the WC–10Ni coatings are summarized in Table 2. The surface topography analysis reveals that the Axial III (HV-APS process) resulted in a significantly smoother surface (Ra = 4.97 ± 0.27 μm; Rz = 29.97 ± 1.68 μm) compared to the conventional A60 torch (APS process—Ra = 6.93 ± 0.49 μm; Rz = 38.72 ± 3.31 μm). The lower Ra and Rz values observed for the Axial III coatings can be attributed to the higher particle velocity in the HV-APS system, which has been reported in the literature [53,54] to be significantly higher than in conventional APS systems due to the high-enthalpy multi-arc configuration and the use of a supersonic nozzle (e.g., 5/16″), promoting more effective splat flattening upon impact, consequently reducing surface irregularities.

The enhanced particle kinematics and the resulting structural densification (lower porosity in HV-APS) not only improved the surface finish but also influenced the hardness measurements. It revealed significant differences between the coatings deposited using the two plasma torch configurations. The coating produced with the conventional single-electrode A60 torch exhibited an average microhardness of approximately 792.14 ± 82.07 HV0.2. This value is comparable to those reported for detonation-sprayed coatings [55], but lower than typical values obtained for coatings deposited by the HVOF process (900–1100 HV) [56]. In contrast, a substantially higher average hardness (1010.26 ± 74.24 HV0.2) was achieved for the coating deposited using the Axial III torch in the HV-APS process.

These results were further confirmed by instrumented indentation testing. The A60 coating exhibited an indentation hardness (H_IT_) of 8871.57 ± 1273.66 MPa, while the Axial III coating achieved a higher value of 10035.96 ± 1788.07 MPa. This increased hardness is likely associated with the improved structural densification and enhanced interlamellar bonding achieved during the HV-APS process. Additionally, the indentation modulus (E_IT_) was evaluated to characterize the elastic behavior. Despite the differences in hardness, both coatings exhibited comparable modulus values, ranging from 216.44 ± 25.37 GPa for Axial III to 221.56 ± 22.08 GPa for A60.

This indicates that while the process parameters significantly influence the resistance to plastic deformation, the intrinsic elastic response of the WC–Ni cermet remains relatively consistent. This observation is particularly relevant when considering the functional performance of the coatings. Erosion resistance tests, conducted at a 90° impact angle, showed that both variants have similar wear rates, despite the higher static hardness of the Axial III coating. The average erosion rate for the Axial III coating was 0.70 ± 0.01 mg/min, while the A60 coating recorded 0.72 ± 0.13 mg/min. In particular, while absolute wear rates were comparable, the Axial III process demonstrated exceptionally high statistical stability, with a standard deviation nearly ten times lower than that of the A60 process. This enhanced consistency confirms that the advanced HV-APS configuration produces a more uniform and predictable microstructure, which is a critical advantage for industrial applications, even if it does not lead to a significant reduction in the total mass loss under the tested conditions. Furthermore, the results obtained are superior to those reported for WC–CrC–Ni coatings deposited by both HVOF and APS processes [57].

### 3.3. Scratch Test and Adhesion Analysis

**Figure 7:**
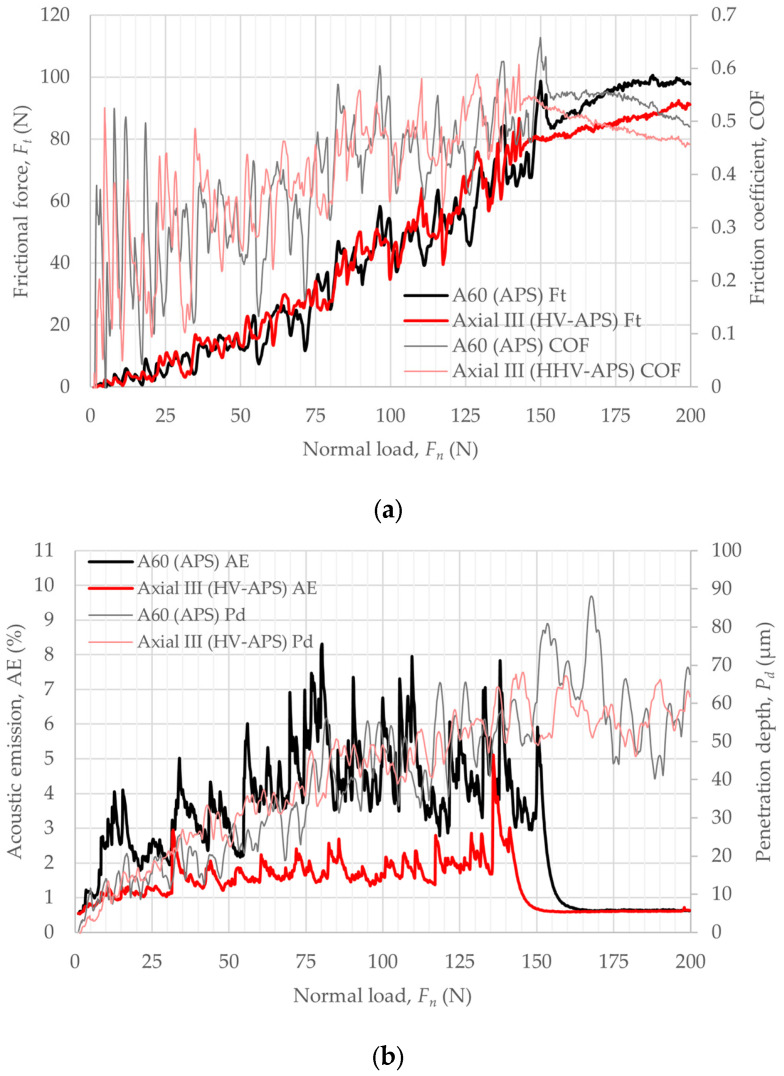
Scratch test results for the WC–10Ni coatings as a function of the normal load (*F**_n_***): (**a**) frictional force (*F**_t_***) and friction coefficient (COF); (**b**) acoustic emission (AE) and penetration depth (*P**_d_***).

The mechanical stability and cohesive strength of the WC–10Ni coatings were evaluated using a progressive scratch test with a maximum normal load (*F_n_*) of 200 N. The recorded signals, including frictional force (*F_t_*), coefficient of friction (COF), acoustic emission (AE), and penetration depth (*P_d_*), are presented in Figure 7.

As shown in Figure 7a, both coating variants exhibit a gradual increase in frictional force with increasing normal load. Throughout the initial stage of the test, up to an *F_n_* of approximately 150 N, the *F_t_* and COF signals display noticeable fluctuations, indicating repeated local damage events occurring during indenter movement across the coating surface. These fluctuations are accompanied by a pronounced acoustic emission activity (Figure 7b), confirming the occurrence of fracture-related processes within the coating structure. The A60 coating exhibits substantially higher AE amplitudes (reaching peaks of 7–8%) and a greater number of acoustic events compared with the Axial III coating (typically below 3% in the same range). According to Miguel et al. [58], such higher AE activity is directly linked to more intense cohesive failure and interlamellar cracking. In the case of the Axial III coating, the lower AE response provides functional confirmation of the improved inter-lamellar cohesion and reduced porosity observed in the microstructural analysis (Section 3.1). This behavior is consistent with the higher densification achieved in the HV-APS process, which may contribute to a lower frequency of crack initiation events under localized loading. Consequently, the Axial III coating shows a reduced tendency for brittle damage events under scratch loading compared with the more porous A60 variant.

A distinct transition in the wear mechanism is observed beyond the threshold of 145–155 N. In this high-load regime, the AE signal for both coatings drops sharply to a near-zero level, and the COF values begin to stabilize while the penetration depth continues to increase. This behavior suggests that fracture-related events become less frequent at higher loads and that the scratch process gradually transitions towards a deformation-dominated wear regime. At this stage, the penetration depth reaches approximately 60–80 μm, indicating an extensive deformation of the coating material under the indenter.

**Figure 8:**
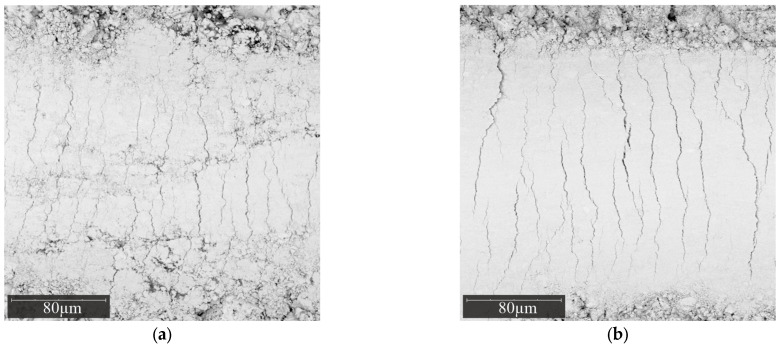
SEM micrographs (magnification 1000×) showing the morphology of the scratch track at a normal load of approximately 184 N: (**a**) A60 (APS) coating; (**b**) Axial III (HV-APS) coating.

The morphological evidence of the damage mechanisms is presented in Figure 8, which shows SEM micrographs of the scratch tracks obtained at a normal load of approximately 184 N. Both coatings exhibit extensive cohesive cracking within the scratch groove, confirming that brittle fracture remains a dominant damage mechanism under severe loading conditions. The observed cracks are predominantly transverse to the scratch direction, which confirms the dominance of tensile stresses behind the moving indenter, a mechanism described by Ghabchi et al. for WC–based thermally sprayed coatings [59]. The A60 coating reveals a dense and irregular crack network, accompanied by numerous secondary cracks that branch out from the main transverse cracks (Figure 8a). In contrast, the Axial III coating exhibits a more regular crack pattern with larger spacing between individual transverse cracks (Figure 8b).

Despite the higher indentation hardness of the Axial III coating (H_IT_ ≈ 10 GPa), extensive transverse cracking is still observed at high loads, indicating that brittle fracture remains an important failure mechanism. However, the significantly lower AE response recorded throughout the scratch test suggests that the HV-APS coating experiences fewer or less severe fracture events during loading. This behavior is consistent with the denser microstructure and improved mechanical properties of the Axial III coating, which enhance its resistance to crack initiation and propagation while not completely eliminating brittle failure under extreme concentrated loading.

### 3.4. Wear Resistance

The tribological performance of the WC–10Ni coatings deposited using conventional APS (A60 torch) and HV-APS (Axial III torch) was evaluated under dry sliding conditions. The results are presented in terms of wear volume, friction behavior, and wear track morphology.

The average wear volume loss for both coatings is shown in Table 2. The coating produced using the A60 torch exhibited a wear volume of 1.38 mm^3^, while the Axial III coating showed a slightly higher value of 1.41 mm^3^. The corresponding specific wear rates were 9.20 × 10^−5^ mm^3^·N^−1^·m^−1^ and 9.40 × 10^−5^ mm^3^·N^−1^·m^−1^, respectively (Table 2). The differences between the two coatings are marginal and fall within the range of experimental scatter, indicating comparable wear resistance under the applied test conditions. The wear volumes obtained are consistent with the values reported for thermally sprayed WC–based coatings in the literature, where wear behavior is strongly dependent on the microstructure and test conditions of the coating [60].

Although the Axial III coating exhibited higher hardness and lower porosity compared to the A60 coating, this did not result in a measurable reduction in wear volume. This suggests that the tribological response is not governed solely by hardness or porosity but rather by the combined effect of microstructural features, including splat cohesion, carbide distribution, crack formation, and the development of a third-body layer during sliding contact.

**Figure 9:**
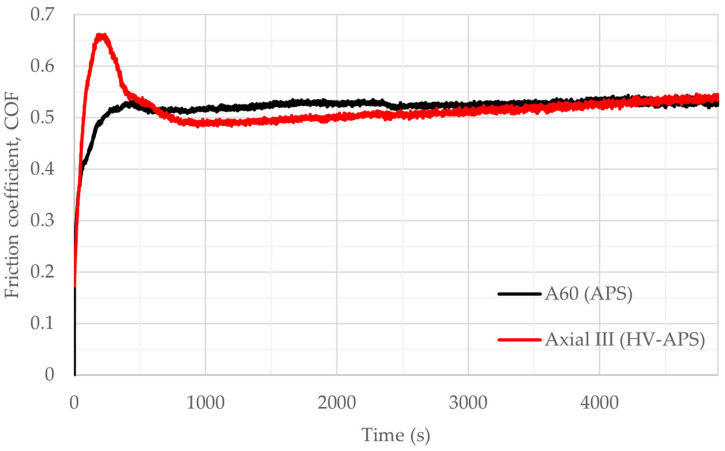
The dependence of friction force on test duration in wear resistance studies of the WC–10Ni coating under friction conditions for both conventionally sprayed by the APS process (A-60 plasma gun) and HV-APS (Axial III plasma gun).

The evolution of the friction coefficient during the testing is presented in Figure 9. Both coatings exhibit a typical running-in stage followed by a steady-state regime. The A60 coating shows a more gradual transition to stable friction conditions, while the Axial III coating exhibits slightly higher initial fluctuations before stabilization. In the steady-state regime, both coatings demonstrate similar coefficients of friction, indicating comparable tribological behavior under prolonged sliding contact. Unfortunately, no directly comparable results have been identified in the literature due to differences in coating systems and testing configurations, including variations in counterbody materials, applied loads, and sliding distances [60]. In particular, the use of a high-hardness WC counterbody in the present study results in a different wear regime compared to tests performed with alumina balls, which are more commonly reported in the literature.

**Figure 10:**
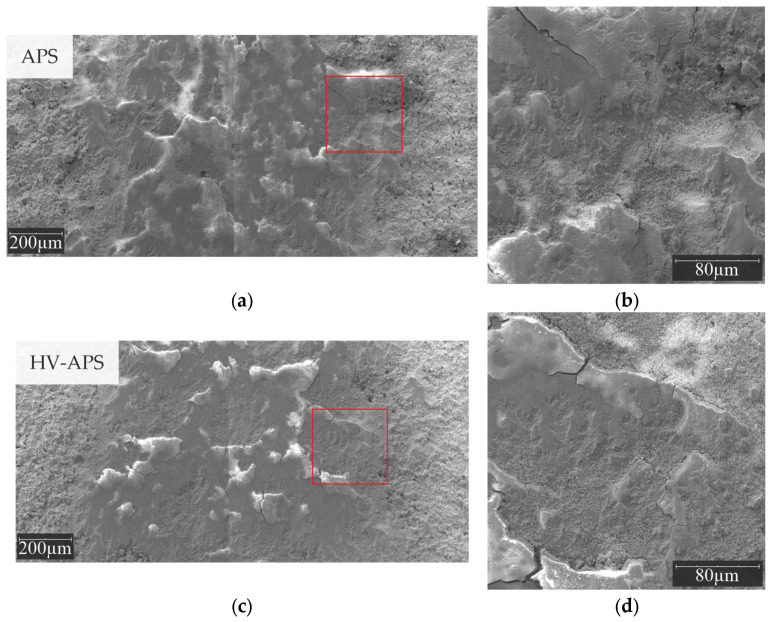
SEM micrographs showing the wear track morphology after ball-on-disk testing for WC–10Ni coatings: (**a**) overview of the combined wear track for the APS (A60) coating; (**b**) high-magnification (1000×) detail of the A60 wear surface of the area marked with a red frame in (**a**); (**c**) overview of the combined wear track for the HV-APS (Axial III) coating; (**d**) high-magnification (1000×) detail of the Axial III wear surface of the area marked with a red frame in (**c**).

Scanning electron microscopy observations of the wear tracks (Figure 10) reveal similar surface morphologies for both coatings. In both cases, worn surfaces exhibit characteristics of abrasive wear and localized microfracture, together with surface smoothing due to plastic deformation and material compaction during sliding. No significant differences in the wear track morphology were observed between the A60 and Axial III coatings.

Despite these subtle differences in the wear track morphology, both coatings demonstrate similar overall wear intensity, which is consistent with the wear volume measurements presented in Table 2. The observed surface features indicate that the wear process is controlled by progressive material removal accompanied by microfracture and debris formation rather than catastrophic delamination.

The presence of hard secondary phases such as W_2_C, which may form during plasma spraying, has been reported to increase brittleness and promote crack initiation in WC-based coatings [61]. The similar wear resistance observed for both coatings despite differences in hardness and porosity suggests that multiple competing microstructural factors govern the tribological response. Although the Axial III coating exhibits improved resistance to crack initiation and lower acoustic emission activity under scratch loading, this advantage does not translate into a significant improvement in sliding wear resistance. This decoupling highlights the differences in the dominant damage mechanisms between scratch-induced deformation and steady-state tribological wear.

## 4. Conclusions

Microstructural investigations confirmed that both APS (A60 torch) and HV-APS (Axial III torch) coatings exhibit a typical lamellar structure characteristic of plasma spraying, consisting of flattened splats, interlamellar boundaries, and porosity. The WC–10Ni coatings showed a thickness of approximately 316 μm for the A60 torch and 205 μm for the Axial III torch. A clear difference was observed in porosity, which decreased from about 12 vol.% for the APS coating to approximately 6 vol.% for the HV-APS coating. Although this represents a significant improvement, the porosity level remains higher than typically reported for HVOF-sprayed WC-based coatings, where values below a few percent under optimized conditions are achievable. For example, optimization of HVOF parameters for fine-grained powders enables extremely dense microstructures, which are more difficult to achieve in plasma processes due to lower particle velocities [5].

XRD phase analysis confirmed the presence of the W_2_C phase in both coatings, indicating decarburization of WC during plasma spraying. This phenomenon is associated with the high thermal input of the process and the formation of brittle carbon-depleted phases [49], distinguishing plasma-sprayed coatings from low-temperature deposition techniques such as cold spraying, where phase decomposition is minimized [51].

The HV-APS process resulted in a significantly smoother surface (Ra = 4.97 ± 0.27 μm, Rz = 29.97 ± 1.68 μm) compared to the conventional APS coating (Ra = 6.93 ± 0.49 μm, Rz = 38.72 ± 3.31 μm). This improvement is attributed to enhanced particle velocity and more effective splat flattening during deposition [53].

Mechanical characterization confirmed the beneficial effect of the HV-APS process. The Axial III coating exhibited a higher microhardness (1010.26 ± 74.24 HV0.2) compared to the A60 coating (792.14 ± 82.07 HV0.2), which is consistent with improved densification and reduced porosity. The higher hardness of the HV-APS coating is attributed to improved structural densification and stronger interlamellar bonding. These values are consistent with the lower hardness range reported for WC–Ni coatings sprayed with HVOF, which can reach values as high as 1338 HV0.3 [7]. For comparison, Nb-modified WC–Ni coatings exhibit a hardness in the range of 980–1032 HV30 [62], indicating that the HV-APS coating approaches the performance level of advanced WC-based composite systems. Instrumented indentation results showed a similar trend in hardness (*H_IT_*), while the indentation modulus (*E_IT_*) remained comparable for both coatings, indicating that the elastic response of the WC–Ni system is relatively insensitive to the deposition process.

Erosion testing at a 90° impact angle revealed no statistically significant differences between the coatings, with comparable mass loss rates of 0.72 ± 0.13 mg/min for A60 and 0.70 ± 0.01 mg/min for Axial III. Although the average erosion resistance was similar, the HV-APS coating exhibited significantly lower scatter in the results, indicating a more uniform microstructure and improved process stability. Despite differences in hardness, the comparable erosion resistance of both plasma-sprayed coatings may result from the presence of decarburization products, which—although hard—increase the brittleness of the structure, favoring cracking at high impact angles [5,6].

Progressive load scratch testing further revealed that the Axial III coating exhibits improved resistance to crack initiation and lower acoustic emission activity compared to the A60 coating. However, this improved resistance under concentrated contact does not lead to a measurable improvement in steady-state sliding wear performance.

Tribological ball-on-disk tests showed nearly identical volumetric wear rates for both coatings, despite differences in hardness and porosity. SEM observations of the wear tracks confirmed similar surface morphologies, characterized by abrasive wear features and localized microfracture, with no significant differences between the coatings.

In general, the results demonstrate that the HV-APS (Axial III) process significantly improves surface quality, hardness, and microstructural uniformity compared to conventional APS (A60). Scratch testing results indicate a higher resistance to crack initiation and a lower acoustic emission activity for the HV-APS coating, suggesting an improved resistance to localized damage under concentrated loading. However, these improvements do not lead to a measurable improvement in steady-state sliding wear resistance, indicating that this type of wear is not directly governed by hardness and porosity alone under the tested conditions. Therefore, HV-APS is more advantageous when high surface quality, mechanical strength, and resistance to crack initiation are required, whereas both coatings exhibit comparable performance under erosive and steady-state tribological loading.

## Figures and Tables

**Table 1:** Plasma spraying parameters of the WC–10Ni coating using the conventional APS process (A60 plasma torch) and the HV-APS process (using Axial III plasma torch).

Process Parameter	Values for A60 (APS)	Values for Axial III (HV-APS)
Spray distance (mm)	100	100
Powder feed rate (g/min)	25	25
Power current (A)	450	3 × 160
Number of torch passes (-)	60	60
Torch traverse speed (mm/s)	1000	1000
Argon flow rate (NLPM)	68	140
Hydrogen flow rate (NLPM)	5	20
Nitrogen flow rate (NLPM)	-	100

**Table 2:** Summary of the properties of WC–10Ni coatings produced by the APS and HV-APS processes.

Parameter	Values for A60 (APS)	Values for Axial III (HV-APS)
Thickness (µm)	316.29 ± 10.05	204.64 ± 12.34
Porosity (vol. %)	11.99 ± 1.73	6.16 ± 0.69
Roughness Ra (µm)	6.93 ± 0.49	4.97 ± 0.27
Rz roughness (µm)	38.72 ± 3.31	29.97 ± 1.68
Microhardness HV0.2	792.14 ± 82.07	1010.26 ± 74.24
Indentation Hardness H_IT_ (MPa)	8871.57 ± 1273.66	10,035.96 ± 1788.07
Indentation Modulus E_IT_ (GPa)	221.56 ± 22.08	216.44 ± 25.37
Erosion rate (mg/min)	0.72 ± 0.13	0.70 ± 0.01
Volume loss (mm^3^)	1.38 ± 0.22	1.41 ± 0.33
Specific wear rate (mm^3^·N^−1^·m^−1^)	(9.21 ± 1.49) × 10^−5^	(9.38 ± 2.19) × 10^−5^

## Data Availability

The original contributions presented in this study are included in the article. Further inquiries can be directed to the corresponding author.
